# Are medical educators in general practice untapped potential to increase training capacity in sexual and reproductive healthcare? Results of a survey in London, UK

**DOI:** 10.1080/17571472.2016.1209875

**Published:** 2016-08-01

**Authors:** Richard Ma, Radhika Shah

**Affiliations:** ^a^ Department of Primary Care & Public Health, Imperial College London, London, UK; ^b^ Goodinge Group Practice, The Goodinge Health Centre, London, UK

**Keywords:** Contraception, professional education, general practice, reproductive health, sexually transmitted diseases

## Abstract

**Background:**

Long waiting times for training in sexual and reproductive healthcare (SRH) including long acting reversible contraception (LARC) might lead to attrition from training programmes, leading to reduced capacity for sexual health services, and reduced access to such contraception for women.

**Setting:**

General practice in London, UK.

**Question:**

Can medical educators in general practice be used as untapped potential to train other health care professionals in sexual and reproductive healthcare?

**Method:**

We conducted an online survey to find out the qualifications, skills and willingness of established educators in primary care in London to train other clinicians in sexual and reproductive healthcare, including LARC.

**Results:**

We received 124 responses from medical educators (10.1% response rate from general practitioner (GP) trainers and 59.0% of clinical supervisors for Foundation Year doctors). 86 (69.9%) had diploma of the Faculty of Sexual and Reproductive Healthcare (DFSRH) qualification and further 18 (14.6%) were interested in obtaining this qualification. Eleven respondents were trained to fit intrauterine contraception only, three for contraceptive implants only and 37 were trained to fit both. 50 (40.3%) of 124 respondents were willing get involved in DFSRH training; 74% of these were willing to teach on any component of DFSRH including LARC.

**Discussion:**

There is a shortage of training places and long waiting list for clinicians who wish to train in SRH. This survey suggests there is a pool of GP educators with skills and experience in SRH and are willing to train others. This can potentially increase the training capacity and improve overall access to good contraception and LARC for women.

## Impact statement

Strategies to reduce unplanned pregnancies and abortions must include timely access to information and wide range of contraception including LARC for women. Improving access to timely care can be accomplished by ensuring there are adequate numbers of healthcare professionals trained to provide SRH and LARC.

## Key messages

The capacity for training in sexual and reproductive healthcare can be increased by using existing GP educators who already have the skills and experience in SRH
Our survey suggests there is a pool of educators who are keen to be involved in SRH training.
Some GP educators have identified barriers to be involved in training others in SRH, some of these such as perceived confidence and competence, can be easily addressed through training.

## Introduction

Many women in the UK access contraceptive services from the primary healthcare team including general practitioners (GPs), practice nurses (PNs) and nurse practitioners. Surveys of women have consistently cited general practice as the main source of routine contraception (43% in 2008/09).[1] According to figures from the Office for National Statistics, 88% of women ‘at risk’ of pregnancy were using at least one method of contraception; the oral contraceptive pill and condom were the most popular methods, used by 38 and 37% respectively.[1] Despite this, abortion rate in England and Wales per 1000 women aged 15–44 in 2011 was 16.5 which is double compared to that in 1970 when the rate was 8.0 per 1000 women.[2]

The National Institute for Health and Care Excellence (NICE) issued clinical guidance on the use of long acting reversible contraception (LARC) in England and Wales in 2005, recommended women requesting contraception should be given information on all methods of contraception, including LARC.[3] However, a survey of over 300 health care professionals working in general practice found that while many endorsed the role of LARC in preventing teenage pregnancy, many saw the lack of skills as a barrier to providing LARC in their practice; misconceptions about side effects of contraception methods were also common.[4]

Doctors in the UK who wish to acquire knowledge and skills in sexual and reproductive healthcare (SRH) might train for the Diploma of Faculty of Sexual and Reproductive Healthcare (DRSRH).[5] Further training is required fit intrauterine contraception (IUC) and sub-dermal implants (SDIs), leading to Letters of Competence in Intrauterine Techniques (LoC IUT) and Letter of Competence in Sub-dermal Implants (LoC SDI) respectively (http://www.fsrh.org/pages/Letters_of_Competence.asp). There is a Letter of Competence in Medical Education (LoC MEd) for those who wish to gain accreditation to train learners in SRH.

Current IUC and SDI training programmes might not be practical due to scarcity of designated clinics in general practice, lack of suitable caseload, lack of trainers in the community as well as other logistical problems such as distance to travel.[6] There is anecdotal evidence of long waiting lists for the practice training component of DFRSH and for LoC IUT/SDI both in and outside London.[7–10] Long waits for training might lead to attrition from the training programmes, resulting in further reduced service capacity with fewer practitioners to meet increasing demand.

General practitioner educators (‘GP trainers’) are accredited to train different types of learners in primary care: GP speciality trainees (GPST), Foundation Year (FY) doctors and diploma of the Faculty of Sexual and Reproductive Healthcare (DFSRH)/LoC IUT/LoC SDI trainees. There is usually an extensive programme developed to ensure that up-to-date teaching methods are utilised and most educators have a postgraduate certification at the end of their training. Accreditation processes are different for specific types of learners so a doctor who can train learners on DFSRH programme might not be able to offer training for GP specialty training; however, GPs who are accredited trainers for GPST have automatic accreditation for DFSRH and IUT/SDI training programmes.

Not all GP trainers are involved in SRH training so there might be a potential pool of GP trainers who could add to current training capacity and ease waiting times. We surveyed this group of GPs in London to ask if they had skills and experience in SRH, and if they were willing to train other doctors in this field.

This survey was done in 2012 under the programme of work for the London Sexual Health Programme (www.londonsexualhealth.org) which was established in 2005 to work on behalf of five London Strategic Health Authorities (SHAs) to lead sexual health commissioning and improve sexual health outcomes in London. The programme recruited five GP LARC champions for each of the five SHAs areas in London (North Central [RS], North West, North East, South West and South East) and a GP Sexual Health Champion [RM] to provide overall strategic leadership. The London LARC Network also provided support for practitioners and commissioners to increase uptake of LARC.

## Method

RM devised a draft algorithm and a pilot survey was distributed amongst a working group which consisted of GP LARC champions for all London sectors, some of whom were also GP educators. After amendments from pilot, the survey was distributed using a web link as the main collector via *Survey Monkey*®. This was distributed amongst local DFSRH, GP, Foundation Year 2 trainers via the London Deanery mailing list, commissioners’ network, Twitter, and personal contacts. The responses were anonymous and apart from an email address which was voluntary and unlinked to survey responses, no personally identifiable data were presented.

We submitted a Freedom of Information request to Health Education England in order to find out the number of active Clinical Supervisors in 2012. The number of GP trainers was obtained via the GMC website (http://www.gmc-uk.org/education/approval_trainers.asp). In 2012, there were 122 active Clinical Supervisors according to Health Education England. The number of GP trainers in London for the year 2012 was not available from GMC website so the nearest available number was for the quarter ending March 2013 which was 621.

We did not seek ethical approval as this was a survey of GP educators’ training activities with respect to sexual and reproductive healthcare and it did not involve patients.

The survey was open from January 2012 until March 2012. Reminders were sent out every two weeks until closing of survey. The results were presented at a London LARC conference in June 2012 to an audience of GPs, nurses and sexual health commissioners.

## Results

There were 171 visits to the survey via the web link collector and a total of 125 respondents completed the survey. One respondent was a nurse so was excluded from the total; 63 were GPST trainers and 72 Foundation Year supervisors. We received a response rate of 10.1% (63/621) from GP trainers and 59.0% (72/122) from FY supervisors.

The respondents’ demographic were: modal age of 50–59, and a male to female ratio of 1:2 (43 to 81, one person skipped the question on gender). The most common main role was GP partner (100 respondents, 80.0%) followed by salaried GPs (20, 16.0%), freelance or locum GPs (3, 2.4%), staff and associate specialist grades and consultants (one each). There was a good distribution of respondents representing each of the five SHA sectors in London (Table 1).

**Table 1.** Respondent demographics.

	Responses (*n*)	%
*Age band*		
Up to 29	0	0
30–39	33	26.4%
40–49	41	32.8%
50–59	46	36.8%
60 or older	5	4%
Total	125	100%
*Gender*		
Male	43	34.7%
Female	81	65.3%
Total	124 (1 skipped question)	100%
*Main job role of respondent*		
GP partner	100	80%
Salaried GP	20	16%
Freelance/locum GP	3	2.4%
Staff Grade/Associate Specialist	1	0.8%
Consultant	1	0.8%
Total	125	100%

The most frequent educator role was as GP speciality or FY2 trainers (50.4 and 57.6% respectively (Table 2)). Main learners were GP specialty trainees and FY doctors (56.0 and 56.8% respectively). These groups were not mutually exclusive because of overlap of these roles and learners, which explained why they did not add up to the total number of respondents. Other learners included medical students, nurses and health care assistants.

**Table 2.** Educator’s status.

	Responses	%
*Educator status (may be more than one type of learner so not mutually exclusive)*		
GP specialty trainer	63	50.4%
Educational/Clinical supervisor for Foundation Year doctors	72	57.6%
Trainer for DFSRH/LoC IUT/LoC SDI	8	6.4%
Other	24	19.2%
*Types of learners within previous 12 months (more than one type of learner so not mutually exclusive)*		
GP specialty trainees	70	56.0%
Foundation year doctors	71	56.8%
DFSRH/LoC IUT/LoC SDI	7	5.6%
Others	26	20.8
None	8	6.4%

Of the 123 respondents (two skipped the question), 86 (69.9%) held DFSRH; out of the 37 who did not, 18 (14.6%) were interested in obtaining it but 19 (15.4%) were not (Table 3). Out of 85 who responded to the question whether they held LoC in IUT and/or SDI, 11 (12.9%) had LoC IUT only, 3 (3.5%) had LoC SDI only, 37 (43.5%) had both, and 34 (40.0%) had neither. Most of those with DFSRH had recertified their qualification (71.4%) compared with LoC IUT (36.9%), LoC SDI (25.0%), LoC MEd (7.1%); 27.4% had not recertified one or more of these qualifications. Of the 38 who did not have DFSRH, 21 (55.3%) had other qualifications or training in SRH including: STI courses (Sexually Transmitted Infections Foundation course organised by British Association for Sexual Health and HIV [BASHH]), old-style Family Planning Certificate (FP Cert), primary qualifications that were not recertified and working experience in genitourinary medicine (GUM).

**Table 3.** Qualifications in sexual and reproductive healthcare.

	Responses	%
*Holder of DFSRH*		
Yes	86	69.9%
No – but would consider	18	14.6%
No – but not interested	19	15.4%
Total	123 (2 skipped question)	100%
*Other qualifications in SRH if no DFSRH*		
Yes	21	55.3%
No	17	44.7%
Total	38	100%
LoC qualification		
LoC IUT only	11	12.9%
LoC SDI only	3	3.5%
Both LoCs	37	43.5%
Neither	34	40.0%
Total	85	100%
*Qualification recertified (not mutually exclusive)*		
DFSRH	60	71.4%
LoC IUT	31	36.9%
LoC SDI	21	25.0%
LoC Med	6	7.1%
None	23	27.4%

50 (40.3%) out of 124 respondents (one skipped the question) were willing to get involved in DFSRH, IUT or SDI training; 25.8% were not and 33.9% were uncertain (Table 4). Of the 50 who were interested, 37 (74.0%) were happy to teach any subject on the DFSRH syllabus; 23 (46.0%) were happy to be a primary or secondary trainer for DFSRH, 27 (54.0%) were willing train GPs for LoC IUT, 28 (56.0%) to train GPs for LoC SDI and a 16 (32.0%) happy to teach on the ‘Course of 5’ which is a training event leading to DFSRH. Of the 73 who felt ambivalent about getting involved in training, 50 (68.5%) said they were not able to commit to the time, 23 (31.5%) did not feel competent or confident enough to train, 17 (23.3%) did not feel there was adequate financial compensation, and nine (12.3%) did not know how they could get involved; other reasons included: not knowing the competencies required, not having recertified primary qualification, already providing training or due retirement.

**Table 4.** Training intentions and barriers of potential educators.

	Responses	%
*Interest in training others?*		
Yes	50	40.3%
No	32	25.8%
Maybe/ Not sure	42	33.9%
Total	124 (1 skipped question)	100%
*Areas of interest (not mutually exclusive)*		
Any subject in DFSRH syllabus	37	74.0%
Becoming a primary or secondary trainer for DFSRH	23	46.0%
Teaching on ‘Course of 5’	16	32.0%
Teaching GPs for LoC IUT	27	54.0%
Teaching GPs for LoC SDI	28	50.6%
Others	4	8.0%
*Reasons for not wanting to train (not mutually exclusive)*		
No time	50	68.5%
Not enough financial compensation	17	23.3%
Unaware of how to get involved	9	12.3%
Not interested	5	6.8%
Do not feel competent or confident	23	31.5%
Other reasons	13	17.8%
*Barriers to GPs/GP trainees to complete training for DFSRH and LoCs (not mutually exclusive)*		
Time to train	90	76.9%
Costs of training	62	53.0%
Unclear training pathways	30	25.6%
Not seen as GP’s role	6	5.1%
Not enough incentives to deliver SRH services	36	30.8%
No barriers	1	0.9%
Structural/organisational barriers	48	41.0%
Don’t know	3	2.6%
Other issues	26	22.2%

The respondents gave a variety of reasons why they themselves, their peers and GP trainees might have difficulties with completing DFSRH or LoC training. 90 (76.9%) stated time as a barrier, 62 (53.0%) stated cost of training, 30 (25.6%) were unaware of training pathways, 36 (30.8%) stated poor incentives to deliver SRH services, 48 (41.0%) stated structural and organisational barriers; 26 (22.2%) cited other reasons including: long waiting lists for practical training, lack of trainers, lack of training facilities and bureaucracy of training pathway.

## Limitations

There might be responder bias in surveys so it is not always possible to verify some of the answers given by respondents; and selection bias so only those who were interested in SRH and training might have been more likely to respond to this survey. However respondents included those who did not have primary DFSRH qualification and also those who were not interested in training others in SRH. Despite not having high response rate from GP trainers (10.1%), we were able to get a high response rate from clinical supervisors for Foundation Year training in London (59%).

We also recognise there might have been missed opportunities to ask further questions about barriers and enablers to obtaining or recertifying SRH qualifications and training others in this field, so a formal qualitative study would be better suited to answer these questions.

## Discussion

Contraception in the UK is largely provided by the primary healthcare team including GPs, practice nurses and nurse practitioners. This snapshot of 124 doctors suggested there were educators in general practice, of whom over two thirds had the prerequisite teaching qualifications as well as experience and skills in SRH, but were unaware they could also deliver training in SRH and LARC. If this finding were to be extrapolated to different training regions across the UK, the capacity for SRH training would increase significantly and shorten the waiting list for training.

There appeared to be barriers for some educators to contribute to overall SRH training capacity. These include internal factors such as: uncertainty of educators’ own competence and knowledge of SRH, lack of understanding about the process of becoming involved in SRH training; and external factors such as: lack of financial and other incentives to train; and uncertainties regarding the process of getting primary qualification in SRH or its recertification. Some educators cited the long waiting list for obtaining qualification in SRH as a barrier to getting the required skills and knowledge to teach it.

Recently, the Faculty of Sexual and Reproductive Healthcare (FSRH) reviewed its training programme and route to obtaining qualifications such as the DFSRH and other LoCs. The recommendations from a report from the FSRH included: revision of the DFSRH regulations to enable both doctors and nurses to train in SRH; adequate access to training centres and consistency of practice for practical training; and modularisation of other practical training such as for IUD, SDI to offer separate accreditation.[11]

In response to the recommendations, FSRH issued a statement of commitment to joint working with Royal College of General Practitioners.[12] The aim is to streamline SRH training for doctors undergoing and after completion of specialist training in general practice. This would help to achieve better access to high quality SRH services, including a wide range of contraception, to the public. The pathway to become a Faculty Registered Trainer (FRT) has also changed to enable general practitioners who are already accredited for education to recognise their skills to train others in SRH.[13]

## Conclusions

Access to contraception and LARC could be improved by ensuring there are adequate numbers of healthcare professionals who are trained to provide good basic SRH care, as well as providing services such as intrauterine contraception and contraceptive implants.

This survey suggested it is possible to increase the capacity for training in SRH by involving GP educators who already have the skills and experience in SRH but who were unaware they could be involved in training programmes. Some of the barriers to increasing capacity of trainers could be addressed easily. There were some GPs who felt ambivalent about getting involved in training because they either were not aware of the formal training pathways for trainees and/or they themselves did not feel confident or competent. These could be addressed by an update course on SRH with a presentation on local training pathways. If this pool of GP educators were mobilised, they could potentially increase the training capacity and improve overall access to good contraception and LARC for women who will benefit.

## Disclosure statement

In accordance with Taylor & Francis policy and our ethical obligation as a researchers, we declare that both of us are general practitioners whose practices receive fees for provision of long acting reversible contraception. Other than this, we report no other potential conflict of interest.
